# Advances in Research, Diagnosis, and Treatment of Neuroendocrine Cervical Carcinoma: A Review

**DOI:** 10.3389/or.2023.11764

**Published:** 2023-11-01

**Authors:** Xiaoyan Ren, Wenjuan Wu, Qiufan Li, Wen Li, Gang Wang

**Affiliations:** ^1^ Chengdu Medical College, Chengdu, China; ^2^ Department of Gynecological Oncology, The Affiliated Women’s and Children’s Hospital of Chengdu Medical College, Chengdu, China

**Keywords:** neuroendocrine carcinoma, neuroendocrine cervical carcinoma, small cell neuroendocrine carcinoma, multimodal therapy, pathology

## Abstract

Neuroendocrine neoplasms (NENs) were classified separately in the 5th edition (2020) of the World Health Organization (WHO) classification of female genital malignancies. Cervical neuroendocrine carcinoma (NEC) is distinguished by its low incidence, high invasiveness, early local dissemination, and distant metastases. The purpose of this review is to outline the achievements in pathology, diagnostics, gene sequencing, and multi-modality treatment of cervical NEC.

## Introduction

**FIGURE 1:**
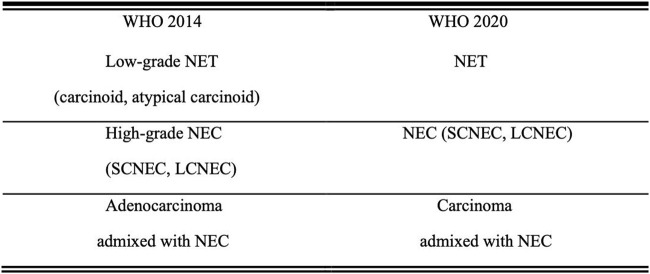
Comparison of the 2014 and 2020 WHO classifications of NENs Abbreviations: NET, neuroendocrine tumor; NEC, neuroendocrine carcinoma; SCNEC, small cell neuroendocrine carcinoma; LCNEC, large cell neuroendocrine carcinoma.

NENs are both aggressive and rare [1]. According to the Surveillance, Epidemiology, and End Results (SEER) database, NETs are most commonly found in the lungs, followed by the gastrointestinal system and pancreas. They can also occur in unknown primary lesions, which may include the uterine cervix [2]. Cervical NEC has a high malignancy rate, a high fatality rate, and a poor prognosis. This type of cancer shares characteristics with NETs and can grow locally or spread to other regions of the body [3]. In Japan, newly diagnosed cervical NEC accounts for 1.6 percent of all instances of cervical cancer. SCNEC accounts for 1.3 percent of this total, while LCNEC accounts for 0.3 percent [4]. This is consistent with existing data that SCCC is more common than LCCC in cervical NEC [5, 6]. As the disease is uncommon in the female reproductive system, the 2020 WHO classification of NENs (Figure 1) categorizes them into two groups: neuroendocrine tumors (NETs) and neuroendocrine carcinomas (NECs), which is different from the 2014 WHO classification of NENs [7]. The terms “low-grade NET” and “high-grade NEC” have been removed from the new categorization. Considering that NECs are frequently linked to adenocarcinoma, the term “Carcinoma admixed with NEC” has been preserved in the 2020 WHO classifications of NENs [8].

HPV infections are related to cervical NEC, specifically with the HPV strains 16, 18, or 35 [9–11]. This is partly because HPV E6 protein breaks down the p53 protein and reduces its production. Moreover, Kasuga found that 72 percent were complicated by high-risk HPV infection (14% HPV16% and 86% HPV18) [12]. In addition, P16INK4A expression is frequently increased in high-risk HPV infections associated with cervical NEC [13]. Argyrophilic cells are created when pluripotent stem cells differentiate into neuroendocrine cells in the mucosal epithelium. These cells may produce endocrine hormones. However, most cervical NEC patients have clinically absent neuroendocrine symptoms, indicating that the tumor either secretes insufficient hormones or produces hormones that are quickly deactivated in the blood [14]. Given the limited treatment options, cervical NEC is prone to early local diffusion and distant metastasis, with high malignancy and death rate and poor prognosis. According to Margolis [15], the risk of death for early-stage small cell cervical NEC (stages IA-IIA) was 2.96 times higher than that for early-stage squamous cell cervical cancer. The prognosis for cervical NEC is quite grim, with a mean recurrence-free survival of only 16 months and a mean overall survival of 40 months [16]. The survival rate over a 5 years period is less than 35%. The likelihood of a poor prognosis is higher for those with advanced stages, high-grade tumors, and a lack of treatment options such as surgery, radiotherapy, or chemotherapy [17, 18]. Additionally, smoking, a cumulative radiation dose (EQD2) of 50 gy, and the absence of brachytherapy have been linked to the recurrence of cervical NEC [19].

## Pathology

Cervical NEC can manifest as an erosive, cauliflower-shaped, or ulcer-like growth. It is usually gray-white or gray-yellow and can penetrate within the cervix to become barrel-shaped, similar to squamous or adenocarcinoma. Under a microscope, the cells have characteristics similar to small-cell lung cancer, but there are some differences. The cells are uniformly small and contain few cytoplasmic components. Tumor cells are often microscopic or intermediate in size and can infiltrate solid sheets or structures that resemble spinal cords. Both mitosis and necrosis are typical and common. However, the nuclei of SCNEC are quite large, densely hyperchromatic, and have unclear nucleoli that are often accompanied by necrosis [20, 21]. Cervical NEC can develop in association with adenocarcinoma or squamous carcinoma, resulting in distal necrosis of squamous or adenocarcinoma cells and invasion of the lymphatic and vascular systems [22].

**FIGURE 2:**
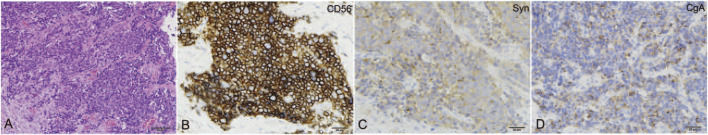
The pathological features of cervical NEC. **(A)** The tumor was composed of small round cells arranged in the nest-like structure (HE, ×200); the tumor cell showed positive for CD56 **(B)**, Syn **(C)**, and CgA **(D)**. Reproduced from ref. 21 with permission, licensed under CC BY-NC 4.0.

According to reports, the neuroendocrine staining agents commonly used are chromogranin A (CgA), synaptophysin (SYN), CD56, and neuron-specific enolase (NSE). However, it has been observed that cervical SCNEC does not always react to these neuroendocrine staining agents, including CgA, SYN, and NSE (Figure 2) [23]. Tempfer discovered that the positive rate of Syn (424/538; 79%), NSE (196/285; 69%), CHR (323/486; 66%), and CD56 (162/267; 61%) in cervical NEC [16]. Various immunological markers have varying degrees of sensitivity and specificity. For example, CD56 possesses a high sensitivity but a low specificity. Consequently, it is preferable to use a combination of IHC markers as opposed to a single marker [20]. For cervical NEC, SYN in conjunction with CD56 is far more dependable. Huang found that the positive rate of Syn and CD56 was 87.75% (82.03%–93.87%, 33.3%), which was higher than the positive rates of Syn and NSE (50.50%–87.68%, 82.7%) and Syn and CgA (53.33%–76.98%, 73.5%) [24]. INSM1 is a new immunological marker that is highly sensitive and specific as a nuclear immune marker [25]. Research has shown that INSM1 has a higher sensitivity than CHR, is equivalent to Syn but lower than CD56, and has a higher specificity than Syn. INSM1 contains unique dots that are not present in typical immune chemicals. For instance, INSM1 is activated when traditional neuroendocrine indicators are difficult to interpret due to obvious squeezing artifacts or acute necrosis in the presence of complement cytoplasmic or membrane staining. As INSM1 is primarily associated with gastrointestinal tumors, it can assist in determining the tumor’s tissue-specific origin as well as the starting point of the metastatic neuroendocrine tumor [26].

## Diagnosis

Cervical NEC can be diagnosed through various methods, including clinical symptoms, radiographic and nuclear imaging, and histopathology. The symptoms of cervical NEC include ectopic neuroendocrine secretion, such as Cushing syndrome, carcinoid syndrome, hypoglycemia, syndrome of inappropriate antidiuretic hormone secretion (SIADH), and hypercalcemia, as well as stomach discomfort. According to Zhao, hyponatremia caused by SIADH is not a good predictor of prognosis and may be used to predict partial recurrence [14]. However, not all patients with cervical NEC will experience symptoms of ectopic neuroendocrine secretion. Instead, most patients may experience abnormal cervical smear, pelvic mass, irregular vaginal bleeding, or postmenopausal vaginal bleeding [27]. Very few cases have been reported where the individual displayed no symptoms. As an invasive illness, cervical NEC may cause distant metastases and systemic symptoms. During a specialist examination, an external cervical tumor may be detected [28], while periuterine thickening or nodules may be found during a triad examination.

Pelvic MRI is better than CT scans for detecting cervical NEC because it has higher soft tissue resolution and can better measure tumor size and local infiltration [29]. However, scar tissue and remaining tumor tissue may have identical signal strengths, affecting MRI accuracy in cervical NEC recurrence. Thus, PET-CT and pelvic MRI complement clinical staging and recurrence. Research shows that the 18F-FDG PET/CT scan is crucial for staging cervical NEC because hematogenous spread can occur early. This scan can detect lymph node involvement or early hematogenous dissemination, changing FIGO staging. Additionally, the 18F-FDG PET/CT scan can detect local recurrence and evaluate therapy response after clinical operations [30].

Immunohistochemistry (IHC) is essential for cervical NEC diagnosis. First, IHC properly replicates the tumor’s origin, which is crucial for diagnosis. Second, IHC can identify squamous and glandular epithelial components, helping determine the cervical NEC’s type [31]. SYN, NSE, CgA, and CD56 are considered classic neuroimmune markers. The majority of cervical NECs contain at least one of these immunological markers.

It is important to differentiate cervical NEC from basal-like squamous-cell carcinoma, undifferentiated carcinoma of the lower uterine segment, rhabdomyosarcoma, and metastatic carcinoma. Cervical NEC can be distinguished from basal-like squamous-cell carcinoma by the fact that the nuclei are not compressed or tightly packed together. Despite this, undifferentiated lower uterine cancer is difficult to recognize due to the presence of neuroendocrine and immunological markers [32]. Rhabdomyosarcoma can be distinguished from cervical NEC by the presence of myogenin and Myo-D1.

## Gene Sequencing

The majority of targeted area sequencing, whole-exome sequencing (WES), and whole-genome sequencing (WGS) are facilitated by Next-Generation Sequencing (NGS) technology. Using these strategies, previously undiscovered genes can be located. Gene mutations are the effective therapeutic targets that can be pursued. PIK3CA, KRAS, PTEN, and TP53 mutations have been identified as the most prevalent cervical NEC mutations [33–35]. Using NGS, wen found a PTEN mutation in one of two cervical NEC patients [36]. Eskander discovered that out of 97 patients with High-grade neuroendocrine cervical cancer (HGNEC), 83 (85.6%) had high-risk human papillomavirus (HPV) strains, mainly HPV 16 and 18. In a genomic analysis of HGNEC, the most common mutations were PIK3CA (19.6%), MYC (15.5%), TP53 (15.5%), and PTEN (14.4%). Gene genomic alterations (GAs) of PIK3CA, TP53, PTEN, ARID1A, and RB1 were associated with HPV. Interestingly, it was found that GAs were more common in the HPV-negative group than in the HPV-positive group. The HGNEC GAs included the PI3K/AKT/mTOR (41.2%), Ras/MEK (11.3%), homologous recombination (9.3%), and Erbb (7.2%) pathways. Notably, among the 97 patients, only 2.1% had a high tumor mutation burden (TMB) with both MSH2 mutations, while 16.5% had an intermediate TMB [37]. However, Microsatellite instability (MSI) is less prevalent than TP53 [38]. Soo further demonstrated that genes in the ATRX, ERBB4, and AKT/mTOR pathways were most frequently altered by WES, signaling that ERBB4-Akt/mTOR inhibitors may be a viable new anticancer treatment option for patients with cervical NEC [39]. In instances of recurrent cervical NEC, the genes associated with DNA mismatch repair (MMR) systems and MYC, TP53, KRAS, and the PI3K-AKT pathway are most likely to be altered [40, 41].

## Multimodality Therapy

Due to the rarity of cervical NEC, treatment options are primarily based on other malignant neuroendocrine tumors outside of the genital tract, as well as common cervical squamous or adenocarcinoma. Currently, multimodal therapy combining surgery, radiation, chemotherapy, targeted therapy, and immunotherapy is the mainstream [42, 43]. However, there is currently no standard treatment for cervical NEC.

Early treatment for malignancies smaller than 4 cm in diameter typically involves radical hysterectomy, regional lymphadenectomy, and postoperative adjuvant therapy. Ishikawa revealed that out of 93 patients with stage I-II high-grade cervical NEC, 88 underwent radical surgery as their initial treatment, while only 5 patients received radiation. In the surgical group, 37 patients received radical surgery and pelvic lymphadenectomy in conjunction with postoperative chemotherapy, 14 received surgery alone, and 25 received surgery with adjuvant or neoadjuvant chemotherapy before the procedure. The mortality hazard ratio in the group exposed to direct radiation was 4.74 (95% confidence interval: 1.01–15.9). The surgical group’s overall survival rate was greater than that of the direct radiation group (*p* = 0.043) [44]. However, Stecklein reported that concurrent chemoradiotherapy is more effective than surgery in treating early-stage cervical NEC with negative lymph node metastasis [45].

For malignancies larger than 4 cm in diameter, some medical professionals recommend using chemoradiotherapy or neoadjuvant chemotherapy before surgery [46, 47]. Research reported that 2018 patients diagnosed with HGNEC at pathological stages IA2 to IIIC2 underwent primary surgery. The 5 years overall survival rate for patients in Stage I, II, and III was 84.9%, 85.7%, and 60.9%, respectively. The Kaplan-Meier survival curves indicated that there was no significant difference in overall survival and progression-free survival between patients who received postoperative chemoradiotherapy and those who only received chemotherapy (overall survival: *p* = 0.77; progression-free survival: *p* = 0.41) [48]. However, it is common to treat this type of cancer with a combination of chemotherapy and radiation. From 1998 to 2002, eight patients with stage III/IV cervical NEC were treated with etoposide and Cisplatin in conjunction with external irradiation and intracavitary brachytherapy at the Columbia Cancer Agency. The three-year survival rate for advanced-stage cervical NEC is expected to be 38%–40% [49]. In addition, protecting fertility is crucial for women of reproductive age. Cervical NEC is a deadly cancer with limited treatment options, so the NCCN does not recommend preserving reproductive function [23].

The guidelines for chemotherapy recommend using either Etoposide-Cisplatin (EP) or vincristine, dactinomycin, and cyclophosphamide (VAC). Studies have shown that EP is less toxic than VAC. In addition, the EP, TP, or TC regimen has been proven effective in specific clinical situations [50, 51]. However, Wang discovered that the combination of etoposide and platinum did not result in an improved overall survival rate after surgery when compared to the combination of platinum and paclitaxel (*p* = 0.71). The univariate analysis showed that patients who received chemotherapy with four or more cycles had a better prognosis than those who received less than four cycles (OS: *p* = 0.01; HR = 6.71; PFS: *p* = 0.02; HR = 5.18). Furthermore, the multivariate analysis indicated that the number of chemotherapy cycles (*p* = 0.02; HR = 0.29) was a prognostic factor for PFS [48]. In addition, chemotherapy can increase the amount of antigens released by immunosuppressive tumor cells upon their death, thereby enhancing the efficacy of immunotherapy. Combining immunotherapy with chemoradiation can have a synergistic effect with less effort. Frumovitz found that the TPB regimen, which combines bevacizumab, paclitaxel, and cisplatin, outperformed non-TPB regimens in terms of progression-free survival and overall survival [52]. The combination of EP and the PI3K inhibitor bez235 significantly slowed the proliferation of HM-1 cells, and cervical NEC cell lines exhibited greater cytotoxic responses due to decreased cell viability and increased apoptosis [53].

Radiotherapy is crucial for treating advanced stages of cervical NEC. Based on the SEER data, Zhang discovered that the median survival time for the surgery group was 44.6 months, while it was 80.9 months for the surgery plus radiotherapy group. However, Radiotherapy should be used with caution when there is no metastasis present [54], as the addition of radiotherapy to surgery did not show significant differences compared to surgery alone (*p* = 0.146) [55].

The recurrence of cervical NEC remains a major troublesome clinical problem. Mabuchi discovered that the initial occurrence of recurrent small cell neuroendocrine carcinoma of the cervix was effectively treated through robot-assisted ultra-radical hysterectomy. While this case firstly exemplifies the security and feasibility of robot-assisted SRH, the extent to which minimally invasive surgery should be utilized in all patients with recurrent cervical cancer remains uncertain [56]. The options for treating relapses of cervical NEC using traditional methods are limited. However, the use of immune checkpoint inhibitors provides hope for patients [19]. Ji discovered that out of the 20 cervical NEC patients tested, 14 (70%) were positive for programmed cell death-ligand 1 (PD-L1) and 15 (75%) were positive for poly ADP-ribose polymerase-1 (PARP1) [38]. The high sensitivity of cervical NEC to PD-L1 and PARP1 suggests that inhibitors for PD-L1 and PARP1, as well as their combination, may provide a novel treatment strategy for cervical NEC. One patient with a second recurrence of stage IIIC1 cervical NEC responded very well to tislelizumab treatment, showing a marked reduction in both supraclavicular lymph nodes and retroperitoneal masses after 3 months of treatment. Thus, Patients with recurrent cervical NEC should undergo molecular testing, such as PD-L1 and MMRs, for personalised treatment.

## Conclusion

Cervical NEC is a rare and aggressive disease with a mean overall survival of 46.3 months [55]. Ectopic secretion is more frequently observed in small-cell lung cancer and gastrointestinal neuroendocrine tumors, whereas the symptoms of ectopic secretion are rare in cervical NEC. The prognosis of cervical NEC is affected by the status of HPV infection, chemotherapy cycles and metastasis. Immunological markers such as Syn NSE, CgA, and CD56, as well as newer markers like INSM1, have shown high sensitivity and specificity in detecting cervical NEC. Currently, there are no ongoing prospective clinical trials for the treatment of cervical NEC; instead, the available studies are mainly retrospective. The choice of postoperative adjuvant therapy varies among oncologists. Adjuvant therapy may involve systemic chemotherapy alone or a combination of therapies, such as concurrent systemic chemotherapy with radiotherapy (CCRT) or sequential chemotherapy followed by radiotherapy. Due to the highly invasive nature of the disease, fertility preservation is generally not recommended in clinical practice. However, further research is necessary to determine the best course of action. In recent years, the development of gene sequencing has provided new targets for targeted therapy, which has helped patients who are experiencing recurrence. In addition to surgery, chemoradiotherapy, and immunotherapy, electric field therapy is being evaluated as an adjuvant treatment for malignancies that have become more aggressive in recent years. Tumor Treating Fields (TTFields) operate on tubulin to suppress spindle formation and tumor cell mitosis [57]. The Phase 2 INNOVATE clinical trial [NCT02244502] confirmed the safety of TTFields combined with weekly paclitaxel in 31 patients with platinum-resistant ovarian cancer (PROC). The progression-free survival (PFS) for TTFields in combination with weekly paclitaxel was 8.7 months, compared to 4.1 months for earlier chemotherapeutic regimens [58]. Therefore, further scientific and clinical research is required to determine if the potential therapeutic benefits of electric field therapy for highly invasive neuroendocrine tumors can be achieved. In the future, additional research and in-depth studies are required to ascertain the biological behavior of these uncommon tumors and develop a treatment strategy that is feasible for them.
